# The Presence of Poultry Influenza Strains in Two Live Bird Markets near the East-West Boundary of Vietnam

**DOI:** 10.1155/2020/1487651

**Published:** 2020-06-04

**Authors:** Quynh Anh Tran, Huong Le Thi, Xuan Thi Thanh Le, Thanh To Long

**Affiliations:** ^1^School of Preventive Medicine and Public Health, Hanoi Medical University, Vietnam; ^2^Vietnam National Centre for Veterinary Diagnostics, Vietnam

## Abstract

The spread of avian influenza virus among Asian countries is becoming a concern after influenza epidemics in recent years. This study is aimed at identifying the subtypes of avian influenza viruses collected from healthy chickens and ducks at two live bird markets in a border province of Vietnam and the Lao People Democratic Republic. Cloacal and tracheal swab samples from 100 chickens and 101 ducks were collected in May 2017. All samples were screened to detect avian influenza virus by real-time reverse transcriptase PCR. Samples that are avian influenza virus-positive were isolated in embryonated chicken eggs, and the subtypes were identified by RT-PCR with the specific primers. The samples positive for influenza virus H5 were sequenced to identify HA and NA genes. The prevalence of avian influenza virus (AIV) among chicken and duck samples was 27.5% (55/200) and 24.8% (50/202), respectively. AIV subtypes identified among 17 samples positive with the hemagglutination test include H3N6, H6N6, and H9N2. Of these 17 samples, 7 duck samples were found to be H6N6, 4 duck samples were infected with both subtypes of H3N6 and H6N6, and two chicken samples were recorded as H9N2. A positive chicken sample with A/H5 contains 99% similarity nucleotide with H5N6 reference strain. Results suggested that while the presence of low pathogenic avian influenza virus is predominant, potential risks of the appearance of high pathogen avian influenza virus in the east-west boundary in Vietnam should be concerned and studied further. Furthermore, prevention activities are needed to reduce such biosecurity threats in Vietnam and other Asian countries.

## 1. Introduction

In the 20th century, millions of people died in four pandemics of influenza virus in 1918 (H1N1), 1952 (H2N2), 1968 (H3N2), and 2009. To overcome the species barrier and direct human infection, avian influenza viruses were thought to be transmitted through intermediate hosts such as pigs to create the necessary variations. However, the H5N1 epidemic in 1997 caused 18 cases in humans with 6 deaths, of which the transmission of the virus was determined to be transmitted directly from poultry to humans [1]. Between 2003 and 2017, the H5N1 avian influenza virus caused widespread outbreaks of poultry and in various territories with at least 860 human cases and 454 deaths diagnosed by the laboratory [2].

Avian influenza viruses are generally species-specific and are primarily pathogenic to poultry. They are widely distributed geographically but rarely cross the species barrier to infect humans and other mammals. However, influenza viruses have fragmented genomes that allow them to be significantly and rapidly altered through the mechanism of drift and exchange of antigens [3]. Therefore, the greater the diversity of viruses, the greater the chance of new virus appearance.

Live bird markets (LBMs) are very popular and an indispensable part of poultry farming in Vietnam and many other developing countries in Asia [4, 5]. In Vietnam, LBMs are found in mostly populated centers, providing fresh poultry that can be purchased for immediate consumption. Live bird markets are home to a large number of poultry, where different poultry types and species are brought together from different geographical locations. Therefore, LBMs are home to many poultry species and contain pathogens from various places. LBMs provide a very favorable environment for the avian influenza virus to exchange and disperse [6, 7]. Previous studies have shown that the poultry markets in Vietnam have many different subtypes of influenza viruses such as H3, H4, H5, H6, H7, and H9 and N1, N2, N3, N6, and N8 [8, 9]. But these studies only take samples at Vietnamese domestic markets. It is concerned that the influenza virus may cross a national border to spread to other regions [10, 11]. Understanding the geographical spread of the influenza virus is important for controlling transmissions among countries and from poultry to humans.

Quang Tri is a province located in the midregion of Vietnam, having boundary with Lao People's Democratic Republic (Lao PDR). Quang Tri is one of three provinces of Vietnam where a road of east-west economic corridor among 5 Southeast Asian countries crossed. In this study, we conducted a sampling at a market near the border with Lao PDR to find out the diversity of influenza virus strains. This study is aimed at identifying the subtypes of avian influenza viruses collected from chickens and ducks at two live bird markets in Quang Tri Province, Vietnam. This study is a part of the project “Reducing Biosecurity Threats from Infectious Diseases with Pandemic Potential in Southeast Asia,” funded by the Asia Partnership on Emerging Infectious Diseases Research (APEIR).

## 2. Materials and Methods

Samples were collected in the Khe Sanh town market and Lao Bao market, Huong Hoa district, Quang Tri Province, Vietnam. Khe Sanh is a central town of Huong Hoa district, meanwhile, Lao Bao is a small town that is border gate with Lao PDR.

Both cloacal swab (CS) and tracheal swab (TS) samples were taken from healthy chicken and duck. The swab samples were collected in May 2017 by professionals from Quang Tri Veterinary Department. 402 swabs have been obtained from 100 chickens (100 CS and 100 TS) and 101 ducks (101 CS and 101 TS) at slaughterhouses in the Khe Sanh town market and Lao Bao market. The first slaughterhouse was selected randomly. At each slaughterhouse, all live chicken and ducks were taken cloacal swab (CS) and tracheal swab (TS) samples until enough 100 chickens and 101 ducks. All chicken and ducks submitted for sale were selected in the Khe Sanh market including 72 chicken and 71 ducks while most of chicken and ducks in Lao Bao smaller market were collected (28 and 30, respectively).

Samples were placed in tubes with viral transport media, kept in a cold box with wet ice, and transported to the National Center for Veterinary Diagnostics within 48 hours of sampling [7]. Samples were then stored in -80°C freezers until further processing. Specimens for virus isolation were placed at 4°C immediately after collection and promptly transported to the laboratory. Sample analysis was performed by the Vietnam National Centre for Veterinary Diagnostics, applying the test method following TCVN 8400:26-2013 [12].

At the Vietnam National Centre for Veterinary Diagnostics, the samples were tested for the presence of influenza type A viruses (M gene detection) using a real-time reverse transcriptase (RT-PCR) method. The threshold cycle applied to determine a positive sample was Ct ≤ 35. At this step, samples having M gene were examined with H5 HA primer/probe pairs. Then, samples having H5 were tested for the N1 and N6 neuraminidase (NA). Next, all positive samples having a threshold cycle Ct ≤ 28 were isolated through the infusion method in embryonated chicken eggs. Samples were inoculated into the allantoic cavity of 9-day-old embryonated chicken eggs and harvested after 72 hours. Eggs were provided by a commercial hatchery and certified as influenza virus-free since 2003. Allantoic fluids were clarified by centrifugation for 10 min at 4°C, and the presence of the virus was determined by the hemagglutination test (HA test). After isolation, the subtypes of the avian influenza virus were identified by RT-PCR with the specific primers for H3, H4, H5, H6, H7, and H9 and N1, N2, N3, N5, N6, N7, N8, and N9. Genes of influenza virus samples positive with H5 were sequenced to identify H and N clades of the influenza virus.

The prevalence of AIV at the individual poultry level was calculated with the total number of AIV-positive individual poultry samples as the numerator and the total number of individual poultry samples as the denominator. Data were presented in numbers and percentages.

This study was approved by the Ethics Committee of Hanoi Medical University (Code number: 104/HMU-IRB dated 15 March 2015). All slaughterhouses were verbally informed and agreed to participate in this study. All information was confidentially kept and used for only research purposes.

## 3. Results

### 3.1. The Presence of Avian Influenza Virus (AIV) in the Swab Samples

Among 402 collected cloacal and tracheal swab samples in two markets, there are 200 swab samples in chicken and 202 swab samples in duck. Among 200 samples in chicken, 144 samples were collected in the Khe Sanh market (72.0%). Among 202 samples in duck, 142 samples were collected in the Khe Sanh bordering market (70.3%).

Among 402 samples, 105 samples were found to be influenza A-positive (26.1%). The prevalence of AIV among chicken and duck was 27.5% (55/200) and 24.8% (50/202), respectively. All samples with AIV were found in the Khe Sanh market.

**Figure 1:**
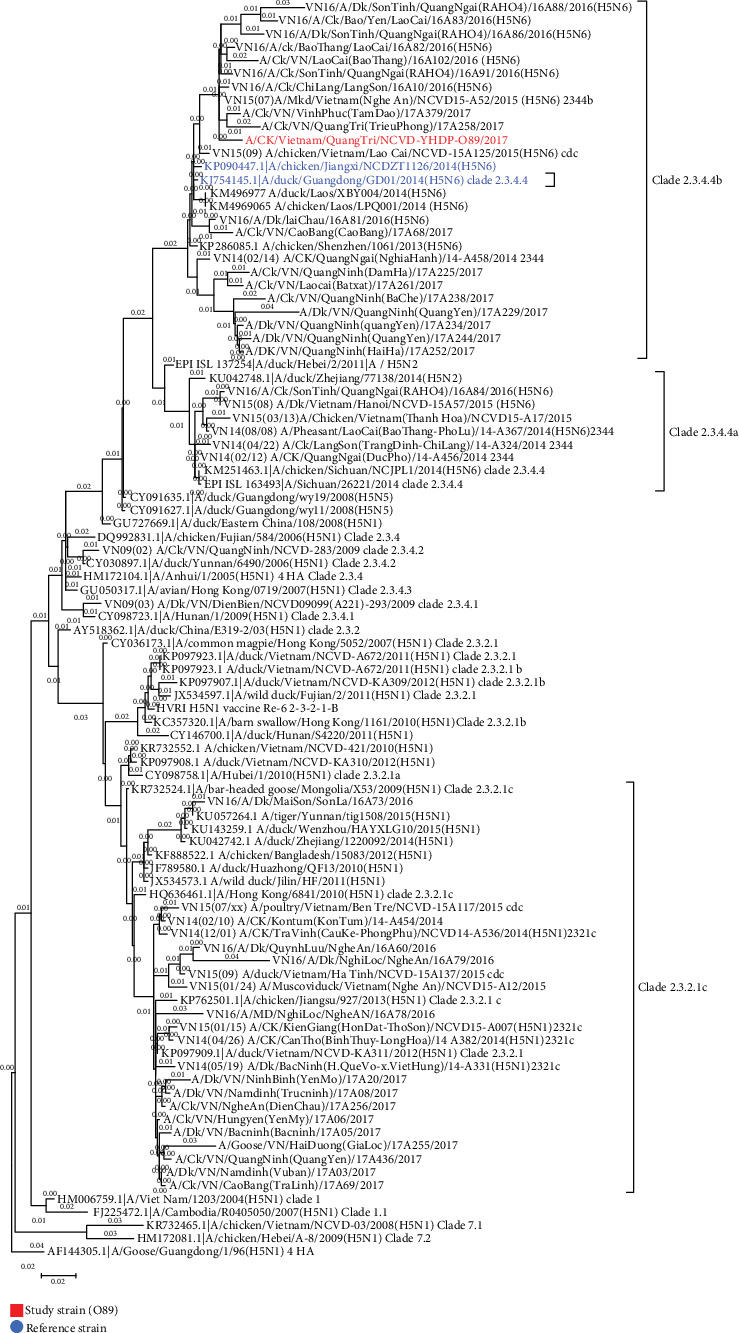
The phylogeny of AIV A/H5 O89. The tree was constructed by neighbor-joining analysis with the use of MEGA 5.3 with 1000 bootstrap replications.

Among 105 samples with AIV (+), one sample from a chicken tracheal swab (coded as O89) was detected to be AIV type A/H5 by rRT-PCR. However, NA subtypes including N1 and N6 were negative. The result of the H5 gene sequence of this sample is presented in Figure 1.

### 3.2. AIV Subtype Identification

Of 105 samples found to be AIV, 54 samples with threshold cycle Ct ≤ 28 were selected to be infused in embryonated chicken eggs to identify AIV subtypes (including the O89 sample with A/H5 positive). After isolation, there were 17 samples positive with HA. The rate of viral isolation was 31.5% (17/54 samples).

HA genes have been identified by RT-PCR including H3 (7 duck samples), H6 (11 duck samples), and H9 (2 chicken samples); no samples were positive with H4, H5, and H7. Regarding NA genes, N2 and N6 have been found (two chicken samples and 14 duck samples, respectively) while no samples were positive with N1, N3, N5, N7, N8, and N9. Subtype of one sample could not be detected.

Table 1 shows AIV subtypes of 17 samples having HA gene. Most of these were cloacal duck swab samples (14/17 samples). AIV subtypes have been identified including H3N6, H6N6, and H9N2. A predominant subtype was H6N6 with 7/17 samples while only two samples belonged to H9N2. Particularly, the H9N2 strain was isolated only from chicken samples while H3N6 and H6N6 strains were found only in duck samples. Four duck cloacal swabs were containing two subtypes of H3N6 and H6N6.

The O89 sample with A/H5 positive at the screening step by rRT-PCR was negative with HA in this step.

Figure 1 illustrates the result of the gene sequence for one chicken tracheal sample (coded O89) that was positive with A/H5 by RT-PCR at the first step. Genealogy tree analysis showed that type H5 in the sample was classified as clade 2.3.4.4b (Jiangxi) (Bi et al., 2015). This study strain has the closest similarity nucleotide to A/chicken/-Vietnam/NCVD15A55/2015(H5N6), A/chicken/Jiangxi/NCDZT1126/2014(H5N6)(NCDZT1126), and A/duck/Guangdong/GD01/2014(H5N6) (GD01) (99%, 97%, and 97%, respectively). The study strain O89 contains basic amino acids at the proteolytic cleavage site of the hemagglutinin protein, LRERRRKR/GLF, identical to the typical cleavage site motif of H5 HPAI virus. The residues of this virus including E190, R220, G225, Q226, and G228 (H3 numbering) are known to be associated with AIV receptor specificity [13].

## 4. Discussion

Although the national surveillance program for avian influenza has been applied widely across the country, it has not reached mountainous and less populated areas [7]. In rural and mountainous areas of Vietnam, domestic poultry is mainly raised in households in a free-range manner. Poultry is transported to LBMs by their owners or poultry sellers. Therefore, LBMs provide a favorable environment for the spreading of influenza virus within the poultry population. Our study is the first study in Vietnam conducted at a bordering district to examine the presence of the poultry influenza strain at LBMs. Findings showed that the presence of the avian influenza virus in the Khe Sanh market was significant, while the avian influenza virus in the Lao Bảo market was not detected. The prevalence of AIV among healthy chicken and duck samples was 27.5% and 24.8%, respectively. This number is slightly higher than the national rate in one previous study, which reported a prevalence of 22.1% influenza virus A positive among 9790 pooled oropharyngeal swabs collected from chicken and duck in live bird markets in 44 provinces throughout Vietnam during 2011-2013 [7]. The prevalence of AIV in poultry samples in the Khe Sanh market is relatively similar to AIV circulation in live bird markets in border areas in Cambodia. A recent study in four border provinces in Cambodia reported that they found that 20.0% of chicken samples and 32.6% of duck samples in domestic markets were positive with AIV by RT-PCR [14].

Study results suggested that the presence of low pathogenic avian influenza (LPAI) was more common than the presence of high pathogenic avian influenza (HPAI) in the study site. After isolation, we found 15 duck samples with subtypes of H3N6 and H6N6 and two chicken samples with the H9N2 subtype. A previous study reported that LPAI viruses were diverse among ducks in different live bird markets across Vietnam, which may be due to domestic trade [8]. In Thailand, besides many studies on high pathogenic avian influenza, some studies have reported genetic characteristics of LPAI in LBM including H7N6, H4N6, and H4N9 [14, 15]. A study in four border provinces in Cambodia also reported that the A/H9 subtype was common in chicken in LBM in this country [14]. Although LPAI was predominant among samples, the common occurrence of the H6N6 strain from duck swab samples is considered as a potential risk of AIV/H5N6 virus reassortant. Previous studies in Lao PDR and China reported that the new H5N6 HPAI virus was reassortment of H5N1 and H6N6 virus [16, 17]. Besides, the detection of different strains of the virus in a poultry body suggested the risks of gene recombination among influenza viruses. In this study, we found four among 14 duck cloacal swabs with two subtypes of H3N6 and H6N6.

The first outbreaks of H5N6 in Vietnam were reported in 2014. This new avian influenza strain was found in the poultry population in Lang Son and Ha Tinh province, located near the border of China and Lao PDR, respectively [18]. Although only one sample from the chicken was classified as H5N6 by RT-PCR and sequencing (99% similarity nucleotide), this finding suggested the risk of a new influenza strain outbreak among the poultry population in another bordering area of Vietnam.

Our study has some limitations. First, all samples positive with AIV were found in the Khe Sanh market; no positive sample was found in Lao Bao. This may be explained by the difference of poultry's sources for sale in two markets that need to be further studied. Second, due to limited resources, we have done sequencing for only H5 samples, not for H3 and H9 to confirm subtypes of these strains.

Despite limitations, our study has detected the presence of various LPAI, a new strain H5N6 HPAI, and potential risks of these strain recombinants in the bordering area. These findings suggested that HPAI is being spread silently across geographical areas. Because LBMs in Vietnam are formed as a traditional culture and could not be banned, intervention programs to improve the biosecurity measures applied in LBM are important. Further studies should be focused on the genetic characteristics of circulating avian influenza strains in these LBMs.

## 5. Conclusions

There was a considerable and diverse presence of low pathogenic avian influenza virus in the Khe Sanh market, more prevalent in ducks than chicken. Potential risks of the recombinant avian influenza virus and the appearance of new strain HPAI are identified. Intervention actions to reduce the risk of an avian influenza outbreak in a border area between Vietnam and Lao PDR are necessary.

## Figures and Tables

**Table 1:** AIV subtype identification by isolation.

Subtypes	Chicken (*n* = 2)		Duck (*n* = 15)		Total
	CS	TS	CS	TS	
H3N6	—	—	2	1	3
H6N6	—	—	7	—	7
H9N2	1	1	—	—	2
H3N6 & H6N6	—	—	4	—	4
Not detected	—	—	1	—	1
Total	1	1	14	1	17

## Data Availability

The data used to support the findings of this study are included in the article.
